# Comment on “Early Prognostic Utility of Gp210 Antibody-Positive Rate in Primary Biliary Cholangitis: A Meta-Analysis”

**DOI:** 10.1155/2020/2453908

**Published:** 2020-01-22

**Authors:** Laura Cristoferi, Pietro Invernizzi

**Affiliations:** Division of Gastroenterology and Center for Autoimmune Liver Diseases, San Gerardo Hospital, Department of Medicine and Surgery, University of Milano Bicocca, Monza, Italy

In the recently published article “Early Prognostic Utility of Gp210 AntibodyPositive Rate in Primary Biliary Cholangitis: A Meta-Analysis”, Huang et al. summarized the published data on autoantibodies anti-gp210, one of the PBC-specific antinuclear antibodies (ANA) with a diagnostic and prognostic values in patients with primary biliary cholangitis (PBC) [1]. Anti-gp210 is indeed the major autoantibody of a family of ANAs directed against proteins of the nuclear pore complex (NPC), but it is widely accepted that the first clear-cut demonstration of the prognostic role of these PBC-specific ANAs comes from an Italian study that focused not only on anti-gp210 but also on anti-NPCs [2–4]. For completeness, we believe that it is important to inform the readers of *Disease Markers* also about this piece of the story.

In particular, we evaluated a unique population of 127 newly diagnosed patients with PBC, naïve to disease-specific therapy, during a 15-year period of follow-up. All patients' sera have been tested for anti-nuclear pore complex (anti-NPC) autoantibodies, both anti-gp210 and anti-p62. Thirty-eight patients' sera showed positivity to anti-NCP antibodies, and 76% of these (*N* = 29) had a reactivity against the antigen at 200 kd (anti-gp210 antibodies). Data have been analyzed based not only on the clinical features but also on Mayo score [5] and specific outcome measures such as time to death, need for liver transplantation, and complication-free survival. Multivariate analysis results showed that prediction models including patients with anti-NCP positivity outperformed significantly the risk of an adverse outcome as compared to both histological stage and Mayo score alone (*p* < 0.39 for death from all causes or need for orthotopic liver transplantation (OLT), *p* < 0.039 for liver-related death or need for OLT). In addition, among patients with early disease (bilirubin at baseline < 1 mg/dL), bilirubin increased to >2 mg/dL in the anti-NCP positive patients (26% vs 5%, *p* < 0.019). Thus, anti-NPC identifies patients that are more likely to experience an unfavourable clinical course and a more rapid disease progression. In the sub-analysis, no differences in clinical indices/outcome measures have been found when considering the reactivity patterns to specific NPC antigens (gp210 and/or p62). Results from our study, and subsequent others, mostly from Japan, were considered solid enough for supporting their use as diagnostic and prognostic biomarkers by the most recent European guidelines for PBC [6].

We hope that our reporting has now filled an information hole, and the readers now know all the solid data available regarding these important biomarkers.
